# Milk Testing by the General Practitioner

**Published:** 1903-05-23

**Authors:** 


### MILK TESTING BY THE GENERAL PRACTITIONER.

As time goes by it becomes more and more evident to the man in general medical practice that he must continually be widening the scope of his attainments in order to retain his position in the ranks of the profession. Nowadays he must be prepared to carry out examinations of urine, sputum, blood, stomach contents, and so forth, the technique of which often involves a considerable amount of time and skill, and it is likely, therefore, that he will be grateful for any hints which may render such examinations simple, rapid, satisfactory, and inexpensive. In the New York *Medical Record* Dr. Keith Shaw describes the various methods which are employed in the examination of milk. It is not necessary to enter into all the details he gives. Some, for instance, the determination of the specific gravity, are sufficiently well known, while others, such as the quantitative estimation of fat, proteids, and other constituents, require considerable time, skill, and expensive apparatus, and are therefore not likely to be frequently carried out by the general practitioner. But there is one examination that requires special notice and that is the estimation of the acidity of milk. There is a close and definite relation between the degree of acidity and the number of bacteria present in a given sample of milk, and it may therefore be taken as an index to the suitability or otherwise of a given sample of milk for human consumption. Milk with an acidity of more than ·16 per cent. should never be used for infant feeding. The limit for ordinary purposes should be ·2 per cent. Acidity of ·3 per cent. is the point of souring. The articles required for making the test consist of an alcoholic solution of phenolphthalein, decinormal sodium hydrate solution, a small glass beaker, a medicine dropper, and a small glass tube holding 10 c.c. and graduated into tenths of 1 c.c. Five c.c. of the milk, which has previously been well shaken, are measured into the beaker, and a couple of drops of the phenolphthalein solution added. The tube is then cleansed and filled to the 10 c.c. mark with the sodium hydrate solution. A small amount of the solution is taken up by the medicine dropper and added drop by drop to the milk which is stirred with a glass rod. As soon as a faint pink colour is obtained the excess of alkaline solution is returned from the dropper to the cylinder and the amount of the alkaline solution used is readily ascertained. The acidity can then be easily determined with the help of the following formula :—

$$\frac{\text{c.c. of alkali used} \times \cdot 009}{\text{c.c. of sample taken}} \times 100 = \text{total acidity.}$$

